# Designing Tunable DNA Condensates to Control Membrane Budding Transformation in Synthetic Cells

**DOI:** 10.1002/advs.202415510

**Published:** 2025-06-27

**Authors:** Nastasja Kaletta, Sophia Burick, Yusuf Qudbuddin, Petra Schwille

**Affiliations:** ^1^ Department of Cellular and Molecular Biophysics Max Planck Institute of Biochemistry Am Klopferspitz 18 82152 Martinsried Germany; ^2^ Department of Molecular Biophysics and Biochemistry Yale University New Haven CT 06511 USA

**Keywords:** budding, DNA nanotechnology, GUV shape transitions, liquid‐liquid phase separation, membrane biophysics, membrane deformations

## Abstract

Wetting interactions between biomolecular condensates and lipid membranes have demonstrated great potential to induce large‐scale membrane transformations in synthetic cells. However, the ability to functionalize existing condensates and control their interactions with membranes is limited, restricting their utility in engineering controlled wetting behavior. Here, fully programmable condensates based on DNA Y‐motifs are introduced to engineer precisely tunable wetting behavior. In contrast to unmodified condensates that show no interaction with membranes, wetting of supported lipid bilayers (SLBs) can be induced by partial cholesterol‐functionalization of DNA nanostructures. Incorporating photoactivatable DNA‐lipid linker enables contact angles to be controlled over a wide range by varying UV exposure times. Furthermore, selective partitioning of small unilamellar vesicles (SUVs) into DNA condensates is demonstrated via programmable surface interactions. In giant unilamellar vesicles (GUVs), membrane wetting of enclosed condensates can be efficiently induced post‐fabrication and results in outward budding. Thus, this work establishes programmable DNA condensates as a powerful platform for fine‐tuned control over membrane‐associated processes in synthetic cells, exceeding traditional approaches such as altering lipid composition or environmental conditions. Finally, the platform provides the possibility to design smart drug carriers for controlled substance delivery and release, and represents a customizable model to study condensate‐membrane dynamics.

## Graphical abstract

In giant unilamellar vesicles (GUVs), membrane wetting of encapsulated liquid‐liquid phase‐separated DNA condensates can be precisely triggered post‐fabrication using photoactivatable DNA‐lipid linkers. On free‐standing membranes, interactions between DNA condensates and the membrane lead to outward budding, resembling exocytosis. The extent of membrane penetration by DNA condensates is influenced by excess membrane area, which can be modulated through osmotic control.

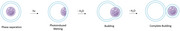

## 1 Introduction

Liquid‐liquid phase separation (LLPS) is a general mechanism cells use to organize their cytosol interior.^[^ ^1^ ^]^ But its physiological relevance is not exclusively restricted to the fluid phase.

In recent years, it has become evident that membrane wetting by biomolecular condensates impact various aspects of cellular organization at membranes, like protein assembly, signaling, formation of autophagosomes and tight junctions.^[^ ^2^ ^]^ In‐vitro LLPS systems have facilitated the investigation of the interplay between membranes and condensates and demonstrated the potential to alter membrane properties. Condensate‐induced membrane transformations can result in ruffled membrane structures, inward or outward budding, tube‐formation, and fission of the membrane.^[^ ^3^ ^]^ Reversely, membranes were shown to also influence condensates with biological significance. Wetting behavior, droplet nucleation and organization can be affected by different membrane properties, like high contents of charged lipids, membrane domains, or lipid packing.^[^ ^3^, ^4^ ^]^ Recently, a photoswitchable azobenzene phospholipid analog that reversible expands membranes of giant unilamellar vesicles (GUVs) was applied to show how endocytosis of glycinin condensates depends on excess membrane area.^[^ ^5^ ^]^ Beyond their fundamental relevance in understanding cellular processes, new applications may result from condensate‐membrane interactions in synthetic or natural cells, e.g., novel drug carriers facilitating intracellular delivery.^[^ ^5^, ^6^ ^]^ However, phase separating systems commonly used in condensate‐membrane interaction studies offer limited flexibility to be adapted or functionalized to particular needs of synthetic cells or material science.

In this context, DNA‐based LLPS systems offer unique advantages due to the programmability, molecular recognition capabilities, and biocompatibility of DNA. DNA nanotechnology was shown to enable the design of nanostructures and reaction networks that replicate biological phenomena.^[^ ^7^ ^]^ Some of them were encapsulated in GUVs as biomimetic compartments and engineered for specific interactions with lipid membranes. Among these are transmembrane elements, such as nanopores, which can be designed for various sizes and shapes,^[^ ^8^ ^]^ as well as DNA signal transducers that exploit the response of DNA elements to specific biomolecules.^[^ ^9^ ^]^ It was also observed that binding of DNA nanostructures has the capability to transform membranes. Either by intentionally designing nanostructures reminiscent of biological membrane‐deforming proteins or as byproduct of DNA‐binding, large‐scale vesicle deformations or membrane tube formation were observed.^[^ ^10^ ^]^ Interestingly, DNA nanostructures can be designed to undergo liquid‐liquid phase separation. In this work, we use DNA Y‐motifs that phase separate due to transient interactions between single stranded overhangs at each end of a Y‐shaped nanostructure.^[^ ^11^ ^]^ In synthetic biology, such phase‐separated Y‐motifs were employed as synthetic nucleus model or artificial cell‐cortex due to hydrogel‐formation in the presence of positively charged lipids.^[^ ^12^ ^]^

In summary, not only do DNA‐based systems offer significantly greater programmability compared to PEG–dextran or protein‐based LLPS systems, but they also enable precise control of droplet–membrane interactions under constant lipid composition and environmental conditions. This represents a highly advantageous feature not only for synthetic biology applications, but also for future work involving theoretical modeling where well‐defined boundary conditions are essential.^[^ ^13^ ^]^

Motivated by the idea to create a membraneless organelle capable of functionalization for applications in synthetic biology, we introduce a Y‐motif‐based liquid‐liquid phase‐separated system specifically designed to controllably induce membrane budding transformations. First, we cholesterol‐functionalized DNA Y‐motifs and observe partial wetting on supported lipid membranes (SLB). This inspired us to design a photoactivatable DNA‐lipid linker to achieve temporal control over the wetting process. Our linker allows for controlled activation and finetuning of wettability depending on UV exposure times. Additionally, we demonstrate how membrane‐interacting DNA droplets can be utilized to concentrate molecules and recruit them to the membrane. Using GUVs as biomimetic compartments, we efficiently induced membrane wetting post‐fabrication. Hereby, we observed droplet‐induced outward‐budding depending on the excess vesicle membrane. Characterizing condensate‐membrane interactions via electrodeformation, we found that excess membrane is efficiently transferred to DNA droplet‐induced budding transition. Thus, by engineering phase‐separated DNA droplets for the emerging topic of condensate‐membrane interactions, we exploit the programmability of DNA to establish a precisely controllable membrane‐interacting system. This even allows for photoinducible vesicle budding reminiscent of exocytosis.

## 2 Results and Discussion

### 2.1 Functionalization of DNA Y‐Motif Condensates with Cholesterol‐Anchor Induces Wetting of Lipid Membranes

**Figure 1:**
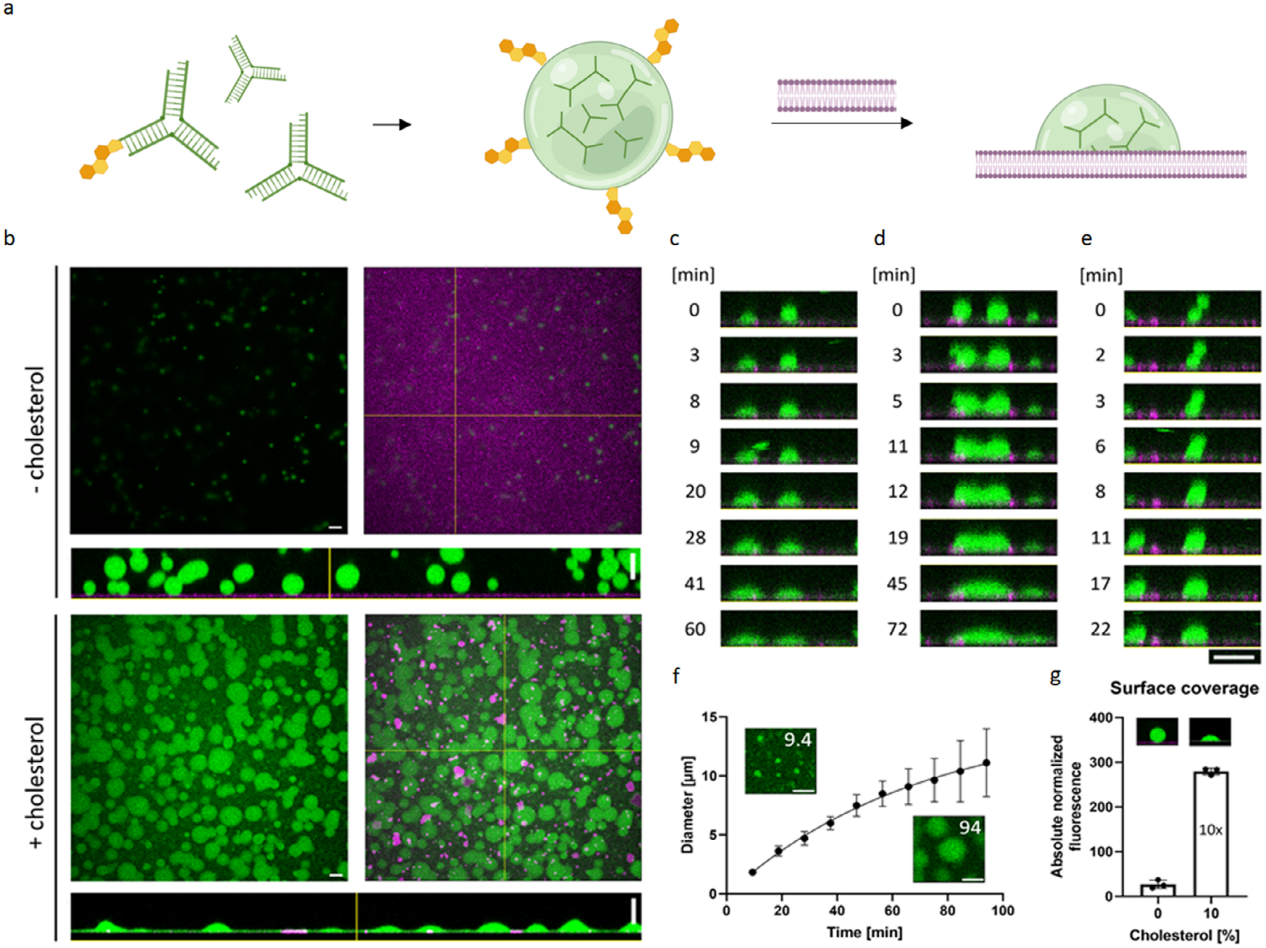
Analysis of wetting and wetting kinetics of cholesterol‐functionalized DNA droplets on supported lipid bilayers (SLBs). a) Schematic view of droplet‐formation from Y‐shaped DNA nanostructures according to Sato et al.^[^ ^20^ ^]^ and membrane‐anchorage of condensates via partial lipidation of DNA nanostructures. b) Confocal images of DNA droplets with and without 10% cholesterol‐anchorage revealing morphological changes in spherical droplets by wetting of SLB membranes. Images were taken after 2.5 h of gradual cool down from 65 °C to room temperature. Green fluorescence intensity corresponds to ATTO 488‐modified Y‐motif strands, magenta indicates ATTO 655‐modified DOPE. Shown is the green channel individually, a merged image, and orthogonal views of droplets in z. Scale bars: 10 µm. c–e) Orthogonal views of z stacks of DNA droplets on SLBs over time. Start of events was set to zero (droplet falling on SLB in c and e). c) Wetting of the SLB membrane. d) Fusion of two DNA droplets on SLB. e) Fusion of membrane droplets with bulk droplets. f) Quantification of diameters of wetted SLB areas by DNA droplets over time for *n* = 10 droplets. Images of droplets on SLBs are shown for two time points (indicated in image in [min]) are shown for comparison. g) Surface coverage of SLBs with DNA. Fluorescence signals of Y motifs targeted to SLB were quantified for three replicates and normalized to DNA‐poor phases in bulk. Data are represented as mean ± s.d. Scale bars: 10 µm.

To induce interactions between lipid membranes and DNA Y‐motif condensates, we introduced cholesterol‐anchors to the DNA nanostructure. Following the design by Sato et al.^[^ ^11^ ^]^ three single‐stranded oligonucleotides were used to hybridize into the Y‐shape and undergo phase separation by transient interactions of single stranded overhangs (6 bases at each arm). One of the strands (Y‐1) was functionalized by a cholesterol‐modification at the 3′‐end (**Figure** **1**a) similar to cholesterol‐modified RNA by Last et al. to induce interactions between condensates and membranes.^[^ ^14^ ^]^

To investigate membrane‐condensate interactions, we first used supported lipid bilayers (SLBs, 70:30 DOPC:DOPG) as model membrane. In contact with SLBs, droplets with 10% cholesterol‐functionalized nanostructures changed their morphology into a truncated sphere indicating partial wetting. A quantification of contact angles between droplet surface and membrane resulted in an average value of 63° for droplets with a cholesterol‐modification (*n* = 9). As a control, droplets without cholesterol‐anchor were formed on SLBs and shown to remain in a non‐wetted state (Figure 1b).

To further investigate the behavior of membrane‐interacting DNA droplets mechanistically, we analyzed their nucleation and growth on SLBs. Using a Peltier stage, samples were heated up to 65 °C to denature interactions of single‐stranded overhangs resulting in a “dispersed state” of the Y‐motifs.^[^ ^11^ ^]^ Starting from this homogeneous solution in which no droplets were visible, formation of DNA condensates on SLBs was induced by lowering the temperature to 40 °C enabling transient DNA‐DNA interactions characteristic for liquid‐like behavior in droplet state. We used fluorescence confocal imaging to track DNA droplets during membrane wetting (Figure 1c–e; Figure S1; Movie S1, Supporting Information). Three events were distinguished in relation to the growth of the DNA droplets. In the first scenario, spherical droplets were formed in bulk and fell on SLBs, resulting in deformation during the time course of wetting (Figure 1c). Membrane‐bound droplets grew either by 2) fusion with other droplets on the membrane (Figure 1d) or 3) droplets from the bulk (Figure 1e), confirming fluidity of DNA droplets at the membrane. Hereby, the contact areas between individual droplets and the membrane increased during viscoelastic relaxation caused by fusion events and time‐dependent wetting. A quantification of the expanding diameters of wetted areas revealed a trend consistent with power‐law (*R(t) ∝ t^α^*, depending on time *t* and growth exponent *α*) during the first 57 min (Figure 1g). Power‐laws describe the two main mechanisms by which condensates grow: 1) Coarsening, where condensates grow into large droplets by evaporation‐condensation of small particles,^[^ ^15^ ^]^ and 2) coalescence, where droplets fuse into larger droplets by collision during Brownian motion.^[^ ^16^ ^]^ However, in contrast to unmodified Y‐motifs, membrane‐interacting droplets remain membrane‐bound over time and at the same place on SLBs during time series (Figure S1; Movie S1, Supporting Information). Molecules interacting with biological membranes commonly display subdiffusion.^[^ ^16^ ^]^ Similarly, LLPS systems binding to a membrane or experiencing restrictions due to a chromatin‐dense environment were also reported to display subdiffusion resulting in reduced growth exponents^[^ ^17^ ^]^ and – in some cases – even arrested droplet growth.^[^ ^18^ ^]^ Therefore, an exponential plateau *(Y = YM‐(YM‐Y0) exp(‐k x)*, described by starting population *Y0*, maximum *YM*, and rate constant *k*, which is inverse of x time units) represents an even better fit ((*R* ^2^ = 0.9972) for membrane‐interacting Y‐motifs, as it considers saturation starting after 57 min more accurately (Figure 1g).

At thermodynamic equilibrium, the DNA fluorescence‐label in the in the Z‐dimension of the membrane was quantified to capture the signal of contact sites between membrane and condensates as well as fluorophore‐labeled Y‐motifs bound to the membrane. Since the contact site with the membrane is comparatively larger for droplets that exhibit wetting, the 10 times higher surface coverage value in samples with cholesterol‐functionalized Y‐motifs confirmed efficient coverage of the membrane surface by DNA droplets beyond quantification at the single‐droplet level (Figure 1g). Notably, we observed that membrane spots frequently appeared in samples with cholesterol‐functionalized droplets (Figure 1b). While no spots or holes were visible in membranes without DNA, spots with membrane fluorescence‐label were observed in the presence of cholesterol‐modified nanostructures even in the absence of DNA droplets (Figure S2a, Supporting Information). This suggests the high concentration of cholesterol‐modified DNA (0.5 µm in 150 µl) as cause while no connection between spots and DNA droplets was observed. A fluorescence recovery after photobleaching (FRAP) experiment was performed with the membranes wetted by cholesterol‐functionalized DNA droplets to characterize the fluidity of the SLBs. The fluorescent recovery was rapid (t_1/2_ = 24.89 s), uniform, and almost complete (> 80% after 130 s), even in the regions wetted by droplets (Figure S2b,c, Supporting Information) suggesting that there are no significant effects from multiple membrane layers or inhomogeneities in the membrane. In addition, droplets neither preferentially attach to nor avoid the spots in subsequent experiments. This further excludes an effect on or by membrane wetting, as it is established in the literature that droplet‐membrane interactions typically lead to a preference for specific membrane phases.^[^ ^4d^ ^]^

Thus, we demonstrate efficient recruitment and membrane‐wetting by cholesterol‐functionalization of DNA Y‐motifs. The fusion and deformation events we observe confirm the fluidity of Y‐motif droplets at the lipid membrane over a time period of 1.5 h. Hence, we engineered a membrane‐less organelle exhibiting liquid‐like properties while partially wetting the membrane.

### 2.2 Designing a Photoinducible DNA‐Lipid Linker for Temporally Controlled Activation of Membrane Wetting

**Figure 2:**
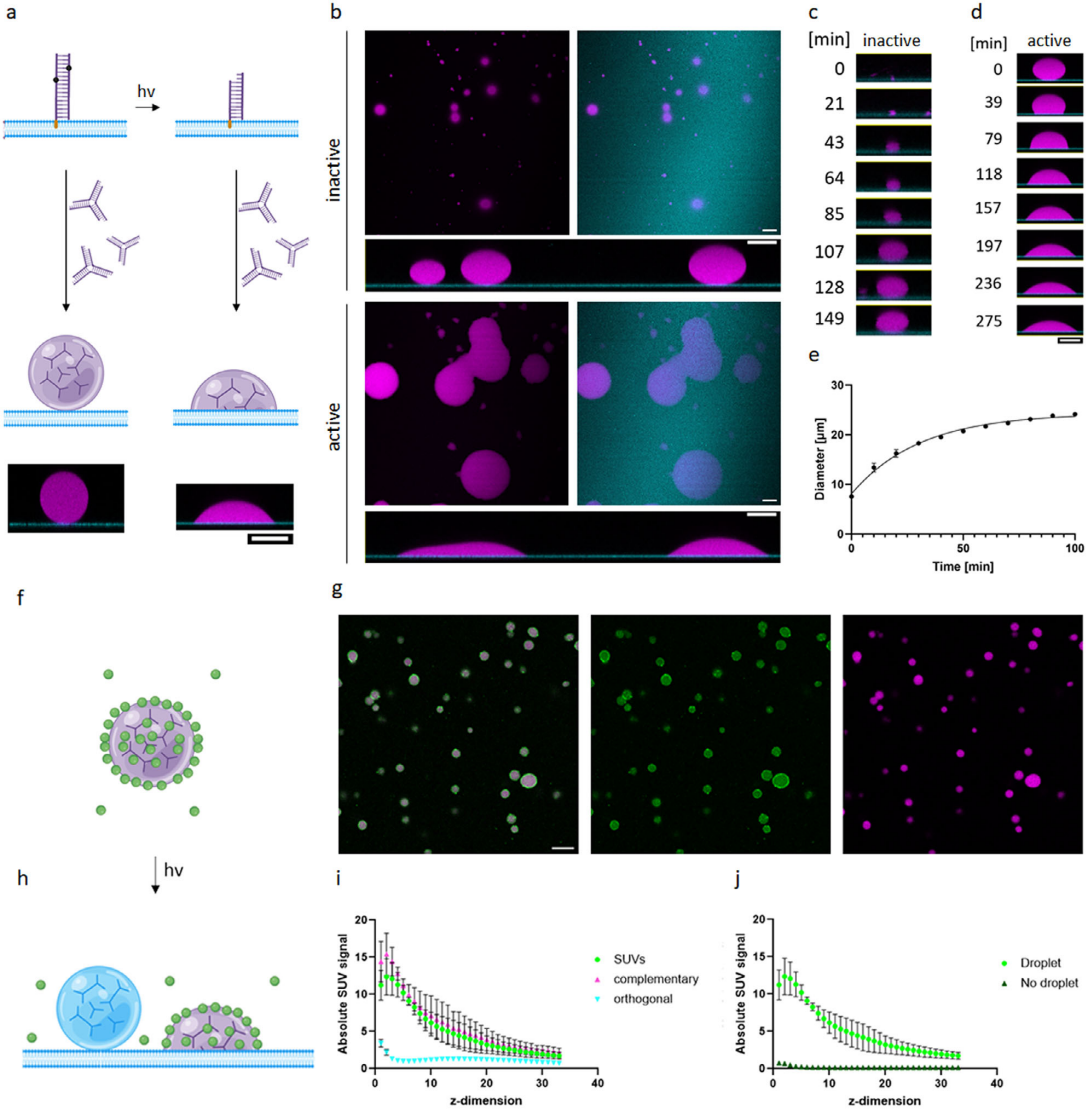
Photoinduced membrane wetting by activation of DNA‐lipid linker. a) Schematic view of a DNA droplet on SLB in the presence of inactivated linker (left) and a DNA droplet in the presence of a photoactivated linker (right). b) Confocal images of DNA droplets with inactivated and activated DNA‐lipid linker revealing morphological changes in spherical droplets upon photoactivated wetting of SLB membranes. Images were taken after 150 min of droplet growth from a dispersed solution on SLBs containing inactive DNA‐lipid linkers and after 300 min incubation after linker‐cleavage. Magenta fluorescence intensity corresponds to ATTO 647‐modified Y‐motif strands, cyan indicates ATTO 565‐modified DOPE. Shown is the magenta channel individually, a merged image, and orthogonal views of droplets in z. c) Growth of a DNA droplet on SLB in the presence of inactivated linker. d) Time course of membrane wetting upon activation of linker. e) Quantification of diameters of wetted areas upon activation of linker over time for *N* = 3 SLBs with *n* = 10 droplets. f) Schematic view of small unilamellar vesicles (SUVs, green) concentrated into a DNA droplet via DNA‐lipid linker. g) Confocal image of small unilamellar vesicles (SUVs, green) concentrated into a DNA droplet via DNA‐lipid linker verified by colocalization. Green fluorescence intensity corresponds to ATTO 488‐modified DOPE. h) Schematic view of small unilamellar vesicles (SUVs, green) concentrated into a DNA droplet wetting a DNA‐lipid linker functionalized SLB membrane. Orthogonal droplets serve as negative control and do not interact with SUVs or SLB. i) Quantification of fluorescence signal of SUVs (green), complementary DNA droplets (magenta), orthogonal droplets (cyan, labeled via ATTO 565‐modified DNA strands), and SLBs (cyan) in z‐stacks (*n* = 3). Interactions between SUVs, complementary droplets and SLBs were possible via DNA‐lipid linker. Z‐stacks were acquired with an optical sectioning set at 0.45 µm intervals. j) Quantification of fluorescence signals of SUVs concentrated into DNA droplets and recruited to SLBs in comparison to SUVs without condensates and unfunctionalized SLBs (*n* = 3). Data are represented as mean ± s.d. Scale bars: 10 µm.

The observation that wetting reliably occurs if membrane interactions are enabled via cholesterol‐anchors motivated us to design a switchable DNA‐lipid linker to actively trigger and temporally control wetting. The linker consists of two DNA strands with photocleavable groups and a 3′‐prime cholesterol‐functionalization for membrane anchoring at one of the strands to allow spontaneous embedding of the linker into lipid membranes. Hybridization of the two strand results in a double stranded piece of DNA. Photocleavable linker within both stands were positioned such that cleavage results in a single stranded overhang at one of the strands. The palindromic sequence of this overhang matches the overhang of Y‐motifs. Hence, linker‐Y‐motif interactions can be activated by UV exposure (365 nm). A sequence of 10 base pairs 5′ of the cholesterol‐anchor and the palindromic sequence was introduced to limit spontaneous denaturation of the double stranded linker which potentially enables undesired interactions between linker and Y‐motif (**Figure** **2**a).

To confirm functionality of the linker, we tested the inactive and active state on SLBs. Y‐motifs formed in distilled water to limit phase separation were added to monitor their growth in the presence of inactive DNA‐lipid linker. Time‐lapse confocal microscopy showed 3D movement of growing droplets on SLBs. Additionally, the de‐wetted state after droplet‐formation confirmed successful shielding of interactions between Y‐motifs and inactive linker (Figure 2b,c; Figure S3a; Movies S2 and S3, Supporting Information). Activation of linkers by exposing SLBs to a UV lamp for 5 min induced wetting of membranes by DNA droplets over time reflected by growing droplet‐membrane contact areas (Figure 2d,e; Movie S4, Supporting Information). Notably, since we functionalized SLB membranes, we drastically reduced the amount of cholesterol‐modified DNA (to 0.5 µm in 20 µl) compared to experiments with cholesterol‐modifier Y‐motifs making membranes less prone to form aggregates (Figure 2b).

We next demonstrated how membrane‐interacting DNA droplets can be utilized to concentrate molecules and recruit them to the membrane. We leveraged control over condensate‐membrane interactions by tagging small unilamellar vesicles (SUVs) to DNA droplets (Figure 2f). Droplet‐formation in the presence of DOPC:DOPG (70:30) SUVs incubated with activated DNA‐lipid linker resulted in the colocalization of SUVs and condensates (Figure 2g). A comparison with DNA droplets bearing orthogonal overhangs showed no enrichment of SUVs, suggesting an additional level of control through the specific accumulation of linker‐modified SUVs in DNA droplets with complementary overhangs (Figure S3b, Supporting Information). Notably, while SUVs partitioned into droplet interiors, accumulation of vesicles at the condensate surface was observed.

To test the possibility to control the recruitment of SUVs to lipid membranes, we produced SUV‐containing DNA droplets in the present of a Y‐motif population with orthogonal overhangs. The solution was incubated for 30 min on SLBs with activated DNA‐lipid linker to specifically induce the membrane‐condensate interactions of only SUV‐containing complementary droplets. Due to their different overhangs, orthogonal droplets served as negative control for no interactions with SUV or SLBs. Fluorescence labeling of SUVs, complementary DNA droplets and orthogonal droplets were quantified in z‐stacks to determine the extent to which they were concentrated at the membrane (Figure 2h,i). SUVs and complementary DNA droplets colocalized well across all Z‐dimensions. The successful induction of SLB membrane‐wetting by complementary droplets resulted in a strong signal for both at the membrane, which gradually weakened with increasing distance. Since the membrane and orthogonal droplets were labeled with the same fluorophore, the lowest Z‐dimensions showed a strong signal attributable to SLB membrane. Because orthogonal droplets could not interact with DNA‐lipid linker and therefore remained in a dewetted state, the signal increased with distance from the membrane, reaching a peak at a height of 6.75 µm above the membrane. Overall, however, the signal of orthogonal droplets was relatively evenly distributed, indicating a strong concentration of SUVs and complementary droplets, which neither qualitatively nor quantitatively correlated with the control of orthogonal droplets.

To quantify the efficiency of targeting molecules to membranes, the signal of linker‐functionalized SUVs on SLBs in the absence was quantified for comparison. Over several micrometers, condensate‐facilitated membrane recruitment results in up to a 43‐fold higher signals in proximity to the SLB, demonstrating highly efficient tagging to the membrane through the system based on programmable DNA interactions.

Taken together, we successfully designed a DNA‐lipid linker to induce and temporally control membrane wetting by DNA droplets via photoillumination. By leveraging the programmability of DNA, we can functionalize our system for the efficient concentration of SUVs into DNA droplets. We demonstrate the possibility to use membrane‐interacting DNA droplets as selective carrier for efficient recruitment of SUVs to the membrane. In our proof‐of‐principle experiment, SUVs are filled with buffer. However, they could be loaded with any molecules to achieve efficient concentration at lipid membranes (Figure 2j).

### 2.3 Membrane Wetting via DNA‐Lipid Linker Enables Fine‐Tuning by Varying the Exposure Time to UV Light

We next quantified contact angles of DNA droplets to investigate the potential of DNA design and the influence of established tuning methods such as lipid composition and salt conditions.^[^ ^3^, ^4^ ^]^

In contact with the membrane, the morphology of phase separated droplets depends on three interfacial tensions: the surface tension between DNA droplet and DNA sparse solution (γ_*ds*_), interfacial tension between membrane and DNA sparse solution (γ_*sm*_), and membrane and DNA droplet (γ_*md*_). At thermodynamic equilibrium, these interfacial tensions determine the cosine of contact angle θ. This relationship is described in the Young's Equation (1):^[^ ^19^ ^]^

(1) $$\cos\theta =\left(\gamma_{sm}-\gamma_{md}\right)\;\;/\gamma_{ds}$$

**Figure 3:**
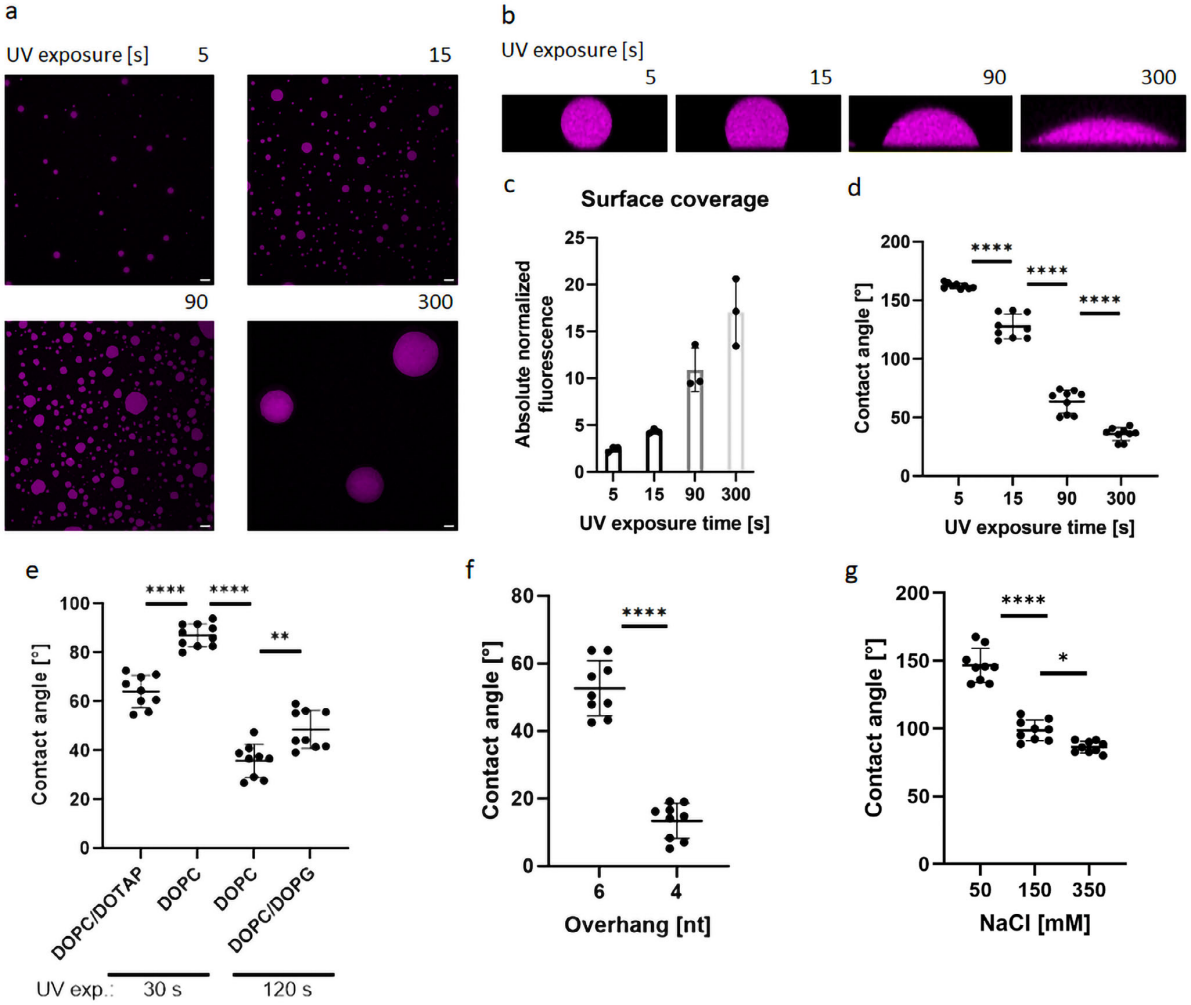
Fine tuning of membrane wetting by varying the exposure times to UV light, lipid composition and alterations in cohesive forces. a) Representative confocal images of membrane contact areas of DNA droplets on SLBs for different UV exposure times (5, 15, 90 and 300 s). Images were taken after incubation at 37 °C over night. Magenta fluorescence intensity corresponds to ATTO 647‐modified Y‐motif strands. Scale bars: 10 µm. b) Representative z stacks of DNA droplets on SLBs for different UV exposure times. c) Surface coverage of SLBs with DNA. Fluorescence signals of Y‐motifs targeted to SLB (*n* = 3) were quantified and normalized to DNA‐poor phase in bulk. d) Statistical analysis of contact angles for three replicates per UV exposure time. e) Statistical analysis of contact angles for three replicates per different lipid compositions: DOPC/DOTAP (70:30), DOPC (100), and DOPC/DOPG (70:30). f) Statistical analysis of contact angles for three replicates per DNA droplets with different overhang lenghts. g) Statistical analysis of contact angles for three replicates per different NaCl concentrations. Data are represented as mean ± s.d. One‐way ANOVA; Tukey's multiple comparisons: *P  ≤ 0.0332, **P  ≤ 0.0021, ***P  ≤ 0.0002, ****P  ≤ 0.0001.

Since γ_*md*_ reflects the interaction strength with the membrane, we intended to tune membrane wetting by varying the UV exposure times (5, 15, 30 and 300 sec). As expected, droplets with shorter UV exposure times displayed significantly less prominent deformations attributed to a partial activation of the linker content and therefore weaker membrane interactions (**Figure** **3**a,b). Significant decrease in membrane interactions for shorter illumination times were also reflected by lower surface coverage by DNA nanostructures (Figure 3a,c).

Contact angles of deformed condensates were determined to quantify wettability. Generally, contact angles smaller than 90° indicate high wettability, while larger contact angles correspond to low wettability.^[^ ^20^ ^]^ After UV exposure of 15 s, the mean contact angle was 128°, indicating low wettability, while an exposure time of 90 s was sufficient to pass the 90°‐criteria for high wettability. Changes in wettability of selected exposure times were statistically significant (Figure 3d). A quantification of the contact angles in samples with different DNA‐lipid linker concentrations reconfirmed tunability via membrane affinity (Figure S4a, Supporting Information).

To further explore the possibilities of fine‐tuning the system, we modified membrane‐condensate interactions via charged lipids. A proportion of 30% Dioleoyl‐3‐trimethylammonium propane (DOTAP), used as a model for positively charged membranes, resulted in significantly smaller contact angles compared to 100% DOPC conditions. In contrast, we observed significantly larger contact angles when using 30% DOPG compared to the condition with 100% DOPC (Figure 3e). Notably, no significant differences in droplet morphology were visible in the dewetted state of DNA droplets on DOPC:DOPG membranes compared to SLBs with 100% DOPC (Figure S4b, Supporting Information). Taken together, these results align with our observation that wetting behavior can be tuned through membrane affinity.

By performing experiments comparing charged membranes with different exposure times (30 and 120 s), we simultaneously demonstrated that the strong influence of the hydrophobic interaction via DNA‐lipid linker remained. In comparison with 100% DOPC control membranes the significantly smaller contact angles in the 30% DOTAP condition is greater than the significantly larger contact angles in the 30% DOPG condition, as the latter condition was exposed to photoillumination for 90 s longer duration. Significant differences in the contact angle of the control conditions with 100% DOPC achieved by different UV exposure times demonstrated that the contact angle can be adjusted via the linker even without electrostatic repulsion by anionic lipids.

We next attempted to control contact angles by making changes in another interfacial energy. In this context, we followed an approach that leverages the programmability of DNA to alter cohesive forces within the droplet as weaker cohesive forces result in stronger wetting.^[^ ^21^ ^]^ Interactions between DNA nanostructures can be modified in various ways. Following a design by Sato et al., we compare our standard design with droplets that exhibit weaker cohesive forces due to shorter overhangs (4 nucleotides).^[^ ^11^ ^]^ Significant differences in contact angles were achieved between different overhang designs (Figure 3f,g). Therefore, we conclude that length of overhang is another strategy to control wetting‐behavior (γ_*ds*_).

In a FRAP experiment for a quantitative analysis of condensate rheology, a slightly higher half‐life was observed for conditions with DNA‐lipid linker compared to conditions without membrane attachment (Figure S5, Supporting Information). In line with a study by Mangiarotti et al., in which enhanced wetting was attributed to the screening of the membrane by the salt we observed an increase in contact angles by reducing NaCl concentrations by several hundred mм (Figure 3g).^[^ ^3b^ ^]^

In summary, we test a broad range of possibilities to fine‐tune wetting‐behavior of DNA droplets including two forms of DNA‐lipid linker‐mediated interactions, different lipid compositions, overhang length, and salt concentration. Hereby, contact angles can be controlled over a wide range via DNA‐lipid anchor by varying UV exposure times. In contrast to conventional approaches to alter wetting behavior of LLPS systems, such as salt concentration or membrane composition,^[^ ^3^, ^4^ ^]^ this constitutes a highly specific parameter to adjust wetting and minimizes side effects on the experimental environment. Under partially extreme conditions, significant differences can be induced by salt concentration and design features like overhang length of DNA nanostructures. However, these strategies do not make the system more controllable but rather more ambivalent. Since DNA droplets do not have a specific affinity for lipid membranes, factors that influence the condensate‐membrane interaction simultaneously affect DNA‐DNA interactions, as both interactions mainly rely on single stranded overhangs of the DNA.

A similar effect is observed with salt concentrations. Facilitated wetting attributed to the screening of the membrane by high salt conditions has already been described in literature.^[^ ^3b^ ^]^ However, since DNA is negatively charged, one would expect high salt concentrations to also weaken repulsive interactions between Y‐motifs, and thus, lead to stronger cohesive forces.

Therefore, with the DNA‐lipid linker‐based system, it is not only possible to achieve a wide range of contact angles, but it is also highly predictable. Since linkers cannot participate into droplets, each linker acts as either a one or a zero regarding membrane‐droplet interactions, depending on whether it is activated or not. Finally, tuning of membrane wetting is achieved while environmental conditions and lipid composition can be kept constant exceeding traditional approaches such as altering lipid composition or environmental conditions.

### 2.4 DNA‐Lipid Linker Allows Induction of Membrane Wetting in GUVs Post‐Fabrication

Cells use liquid‐liquid phase separated droplets to organize and up‐concentrate molecules in so‐called “membraneless organelles”.^[^ ^1^ ^]^ To explore condensate‐membrane interactions in a more cell‐like environment, we used giant unilamellar vesicles (GUVs), as synthetic cell compartments. DNA‐lipid linkers and Y‐motifs were encapsulated in POPC (1‐palmitoyl‐2‐oleoyl‐sn‐glycero‐3‐phosphocholine) GUVs via the inverted emulsion transfer method.

**Figure 4:**
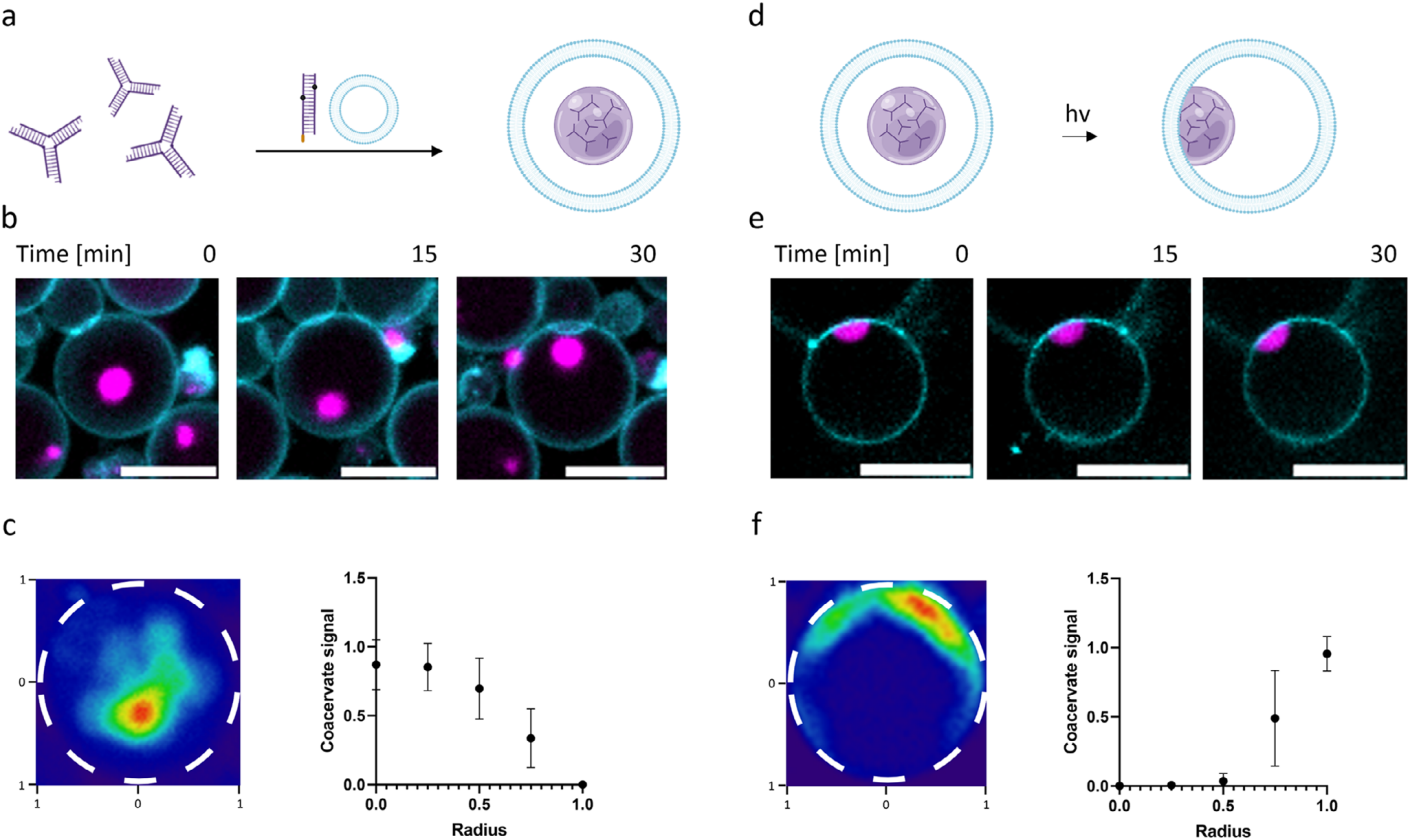
DNA‐lipid linker allows controlled membrane‐wetting in GUVs post‐fabrication. a,d) Schematics outlining encapsulation of DNA droplets and DNA‐lipid linkers in GUVs (a) and vesicle membrane wetting by a DNA droplet upon UV exposure (d). b,e) Representative confocal images from a time‐lapse measurement showing 3D Brownian motion of DNA droplets before UV exposure (b) and membrane wetting upon activation of DNA‐lipid linker (e). Magenta fluorescence intensity corresponds to ATTO 647‐modified Y‐motif strands, cyan indicates ATTO 565‐modified DOPE. Scale bars represent 10 µm. c,f) Representative heat maps and radial plots indicating droplet positions over time (*n* = 30). Radii were normalized from 0 to 1 and standard deviations indicated.

After droplet formation, we performed time‐lapse imaging to track the movement of condensates. In the case of inactive DNA‐lipid linker, droplets diffused randomly in vesicle interiors without prolonged contacts with the membranes (**Figure** **4**a,b; Movie S6, Supporting Information). Droplet positions over time were visualized in heatmaps of individual GUVs by overlaying droplet positions in z and from all time frames and summing up fluorescence signals. In addition, droplet position distributions were quantified in radial plots as function of the vesicle radius to confirm 3D diffusion in vesicle interiors. (Figure 4c).

In contrast, activation of DNA‐lipid linkers resulted in membrane wetting by DNA droplets (Figure 4e). Upon UV exposure, droplets remained attached to the vesicle membrane, showing 2D diffusion along the membrane surface throughout an observation period of 30 min (Figure 4f). Considering there was no diffusion of droplets back into the bulk GUV solution, this suggests efficient recruitment of DNA droplets to vesicle membranes and strong condensate‐membrane interactions.

In summary, we efficiently encapsulate DNA droplets in GUVs. Using the DNA‐lipid linker, strong condensate‐membrane interactions are induced post‐fabrication, leading to wetting at the inner membrane leaflet.

### 2.5 DNA Droplet Interactions with Free‐Standing Membranes Result in Outward Budding

**Figure 5:**
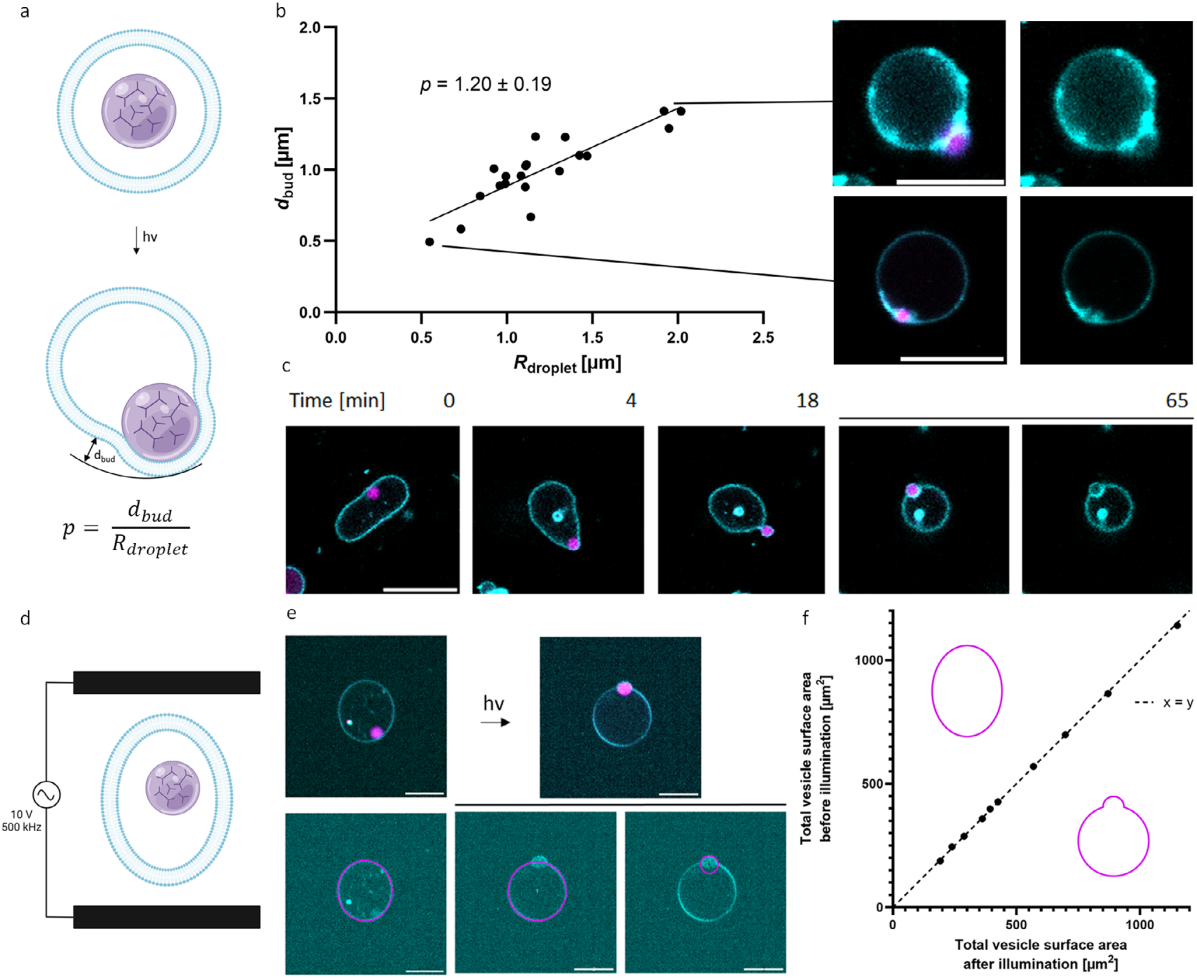
DNA droplet‐membrane interactions result in outward budding dependent on excess vesicle membrane. a) Schematic of photoinduced membrane budding by DNA droplets in an osmotically deflated vesicle. Penetration depth *p* can be calculated by distance *d* between edge of vesicle and bud divided by droplet radius *R*. Diameter d and formula indicated in schematic. b) Bud diameter *d* as function of droplet radii *R* (*n* = 20). Average penetration depth p of GUVs with an initial osmotic pressure from 746.67 ± 2.87 raised to 994.33 ± 2.49 mOsm kg^−1^ indicated in plot. Confocal images of droplet‐induced buds are shown for smallest and biggest bud. Magenta fluorescence intensity corresponds to ATTO 647‐modified Y‐motif strands, cyan indicates ATTO 565‐modified DOPE. c) Interesting time points from time‐lapse imaging of photoactivated complete budding. d) Schematic of DNA droplet‐containing GUV in alternating current (AC) field. e) Confocal images of GUVs in electric field before and after illumination with a 405 nm laser (top). Elliptic and circular fits of vesicles and budding condensate used to determine vesicle surface areas (bottom). An initial osmolarity of 478 mOsm kg^−1^ was elevated to 602 mOsm kg^−1^. By summing up vesicle and droplet‐membrane interface segment, potential alternative membrane transformations are excluded. f) Data fall at a y = x line confirming all excess membrane is transferred to droplet‐induced budding transformation *R* ^2^ = (0.9997). Calculated surface areas before (y‐axis) and after (x‐axis) laser irradiation indicated as 2D image in plot. Each point corresponds to an individual droplet‐vesicle pair (*n* = 10). Scale bars: 10 µm.

In vitro reconstitutions of membrane‐interacting condensates have revealed various membrane transformations.^[^ ^3^, ^22^ ^]^ These observations are typically made in osmotically deflated vesicles, enabling notable membrane deformations. To obtain prominent visible effects at the vesicle membrane, deflation of GUVs was induced by evaporation of the exterior solution. A volume loss of ≈33% resulted in a raise of the initial osmotic pressure from 746.67 ± 2.87 to 994.33 ± 2.49 mOsm kg^−1^. Upon photoactivation, we observed outward budding of encapsulated DNA droplets at the contact site of droplet and membrane. To quantify budding, the degree of the penetration of DNA droplets in GUVs was calculated. Penetration depth *p* describes distance *d* from the edge of the vesicle to the edge of the condensate‐membrane contact area normalized by the droplet radius *R* (**Figure** **5**a):^[^ ^23^ ^]^

(2) $$p=\frac{d_{bud}}{R_{droplet}}$$

In contrast to Dietrich et al. who studied engulfment of particles, *p*‐values of 2 in our encapsulated system correspond to complete budding, while values between 0 and 2 reflect partial budding. The larger the DNA droplet‐condensate system, the greater the measured distance *d*. However, when normalized by the droplet radius *R*, we obtained an average p‐value of 1.20 ± 0.19 (Figure 5b). A quantification of the influence of linker concentration on penetration depth revealed significant differences in *p*‐values. From this, we conclude that a certain interaction strength is needed to efficiently bind and deform GUV membranes (Figure S6a, Supporting Information).

Interestingly, we also observed that some droplets were completely enclosed by membrane. Since budding was reported to depend on excess vesicle area, we hypothesized that complete budding occurs in very deflated GUVs.^[^ ^5^ ^]^ Hereby we adhere to the terminology of Jülicher and Lipowsky, which defines complete budding as a distinct constriction from the vesicle, forming two spherical segments without full separation, in contrast to vesicles that undergo definitive fission.^[^ ^24^ ^]^ Since the origin of these droplets was unclear, they were not included in the quantification in Figure 5b.

To confirm that fully membrane‐enclosed droplets result from complete budding, we performed time‐lapse imaging of the budding process. Hereby, we focused on highly deflated GUVs and induced droplet‐membrane interactions by a short UV exposure time of 15 s. Indeed, time‐series verified that complete budding occurs in very deflated GUVs (Figure 5c; Movie S6, Supporting Information). 85% of vesicles we imaged were classified as partial budding and 15% of vesicles were classified as complete budding (Figure S6b, Supporting Information). Notably, we observed enhanced stickiness of vesicles after UV exposure (Figure S7a, Supporting Information). In line with vesicles that began to stick together after contacting, we observed that daughter vesicles remained in contact with the mother vesicle after budding transformation.

To reduce stickiness and out rule the effect of DNA‐lipid linker interactions observed in a study by Parolini et al., we incubated the samples with DNase I.^[^ ^25^ ^]^ The phenotype of the vesicles after photoillumination changed significantly after incubation with DNase I, which is evident from the smaller contact points between the GUVs, making them appear rounder. However, some stickiness remains, and we exclusively document complete budding events without fission (Figure S7c,d, Supporting Information).

Our observations suggest that the excess vesicle membrane generated by osmotic deflation goes to the DNA droplet‐membrane interface to form buds. However, droplet‐induced budding can potentially compete with a variety of membrane transformations to store excess membrane.^[^ ^5^, ^26^ ^]^ Deformations and tubulations of vesicle membranes were reported to even be promoted by binding of cholesterol‐modified DNA nanostructures.^[^ ^10b,c^ ^]^ To precisely characterize the physical impact of photoactivated droplet‐binding on the membrane, we studied changes in vesicle surface area via the recently employed electrodeformation method.^[^ ^5^ ^]^ Hereby, an alternating current (AC) field is applied to deform vesicles into prolate shape and release membrane fluctuations allowing for accurate quantification (Figure 5d).^[^ ^27^ ^]^

To achieve a response to the electric field, we prepared Y‐motif‐containing vesicles with a lower initial osmolarity of 478 mOsm kg^−1^. The osmolarity was then elevated to 602 mOsm kg^−1^ by diluting vesicle pellets in a NaCl‐free glucose solution. Volumetric images of electro‐deformed vesicles were acquired with a spinning disk confocal microscope. Condensate‐membrane interactions were induced by irradiation with the 405 nm laser line for 1 min in the absence of the AC field. Vesicles which developed droplet‐induced buds were imaged under the electric field. Equatorial planes were selected manually from the volumetric images for both, the vesicle and the buds to fit them with ellipses for vesicles and circles for the buds (Figure 5e).

By considering membrane segments of the vesicle and the area in contact with DNA droplets, surface area of alternative membrane transformations is excluded. Membrane stored in, e.g., tubes, would result in a lower surface area after photoillumination. The y = x line depicts that all of the excess membrane area due to deflation is transferred to the DNA droplet induced buds (*R* ^2^ = 0.9997, Figure 5f).

Therefore, we conclude that outward budding induced by the adhesion energy of DNA droplet‐membrane interactions is the most favorable membrane transformation to store excess vesicle surface area.

## 3 Conclusion

In our study, we demonstrated the successful implementation of controllable phase‐separated DNA Y‐motifs to induce vesicle budding reminiscent of exocytosis. While DNA Y‐motifs usually do not wet membranes, we synthetically generated membrane‐interacting DNA condensates via cholesterol‐functionalization. We exemplify how membrane‐interacting DNA droplets can be utilized to efficiently concentrate biomolecules and recruit them to the membrane. Furthermore, we achieved precise temporal control and fine‐tuning over membrane wetting and budding processes by engineering a photoactivatable DNA‐lipid linker. The possibility to control wetting behavior renders this system a variable membrane‐less organelle, offering flexibility of adaptation to particular features of synthetic cells, but also to applications in materials science or medicine.

In recent years, the biological significance of membrane wetting by biomolecular condensates in cellular organization, protein assembly, signaling, formation of autophagosomes and tight junctions has become evident.^[^ ^2^, ^18^, ^28^ ^]^ Due to experimental challenges of researching liquid‐liquid phase separation in vivo, in vitro LLPS systems have emerged as important model systems.^[^ ^3^ ^]^ Besides their relevance in elucidating fundamental phenomena, in vitro reconstituted condensates may also have great potential for biotechnological or biomedical applications, e.g., as synthetic organelles in artificial cells or as drug carriers.^[^ ^3^ ^]^ However, many protein‐based LLPS systems are limited in their adaptability and customizability. Therefore, we here exploited the programmability of DNA and established a controllable membrane‐interacting system.

Our DNA‐lipid linker may in the future be applied in synthetic cells to mimic cellular processes, such as endocytosis or exocytosis. Hereby, temporal and spatial control over biomolecular membrane interactions is crucial to ensure effective responsiveness to cellular needs. Hereby, DNA nanotechnology also offers further customizability. Leveraging the programmability of DNA, we functionalize our system for the efficient concentration of small unilamellar vesicles (SUVs) into DNA droplets. We successfully demonstrate the potential of membrane‐interacting DNA droplets as selective carrier for efficient recruitment of SUVs to the membrane. While SUVs used in our proof‐of‐principle experiment are filled with buffer, they could be loaded with any molecules to achieve enhanced cellular organization via efficient concentration at lipid membranes.

In our system, spatial control is still limited since membrane wetting happens at a homogeneous membrane. However, in phase separated membranes, DNA‐lipid linkers could be used to target condensates to specific domains for advanced cellular organization. The introduction of membrane organelles harboring different DNA‐lipid linkers may be a superior mimicry for intracellular trafficking. DNA‐lipid linkers located at synthetic autophagosomes could allow for the up‐concentration of specific molecules and degradation of condensates in a second step. Indeed, degradation of condensates via autophagy is regulated by wetting in living cells.^[^ ^2d^ ^]^ Moreover, with regard to the potential design of a synthetic nucleus, a DNA‐based LLPS system provides a tool to generate offspring cells harboring genetic information. Coupling of DNA segregation and cell division can be achieved by combination with DNA droplets undergoing fission. Fission of DNA droplets has been achieved by the cleavage of linker connecting two populations of Y‐motifs with incompatible overhangs.^[^ ^11^, ^12^ ^]^ Furthermore, the programmability and specificity of the interactions could enable sequential droplet fission and the division of multiple generations of daughter compartments.

In drug targeting applications, the ability to induce exocytosis‐like processes could be utilized to deliver therapeutic agents directly into cells. The budding transformation we show can be triggered by a very short illumination time of 15 s. While we cannot completely exclude photodamage on biomolecules, antioxidants and radical scavengers could be added to the system. Alternatively, the possibility to customize the DNA nanostructure allows for alternative modifications, such as proteins or aptamers, with the potential to induce cell‐specific interactions and endocytosis. Desirable characteristics of Y‐motifs as drug carriers are the biocompatibility of DNA and the possibility to up‐concentrate and protect substances like RNA for delivery.^[^ ^7^, ^11^ ^]^ In fact, morphogen release of Y‐motif condensates has been studied in organelle cultures and early embryos in a study by Afting et al.^[^ ^29^ ^]^

To summarize, we here designed and implemented a fully programmable DNA condensate‐based system to gain spatiotemporal control over condensate‐membrane interactions. Our results highlight the potential of DNA‐based LLPS systems as versatile tool for engineering membrane dynamics in synthetic cells, but also beyond. Programmable budding as shown here promises future applications in biotechnology, materials sciences, mathematical modelling and biomedicine.

## 4 Experimental Section

### Annealing of DNA Droplets

Oligonucleotides were purchased from Eurofins (IA, USA, purification of unmodified oligonucleotides: standard desalting, purification of modified oligonucleotides: HPLC Purification), dissolved at 100 µм in milliQ water and stored at −20 °C for further use. Sequences for DNA strands are provided in Table S1 (Supporting Information).

Strands of the Y‐motif were mixed in an Eppendorf PCR tube at 5 µм in a buffer for condensation (20 mм Tris‐HCl, 350 mм NaCl, pH 8.0). To induce condensation via ionic strength, equal molar ratios of oligonucleotide stocks were mixed to anneal. For the introduction of membrane affinity, strand Y‐1 was substituted with different percentages of a cholesterol‐modified strand. Oligonucleotides were annealed on a Thermal Cycler (Bio‐Rad, CA, USA). Samples were heated to 85 °C and cooled down to 25 °C at a rate of −1° per minute.

### Supported Lipid Bilayer (SLB) Preparation

SLBs were formed via vesicle fusion onto a hydrophilic support. Coverslips were rinsed with ethanol and distilled water and dried under a N_2_ stream. Oxygen plasma was used to surface‐etch coverslips (30 s at 0.3 mbar, Zepto, Diener Electronics). Lipids were dissolved in chloroform in a glass vial, and dried under a gentle N_2_ stream. Lipids were re‐suspended in SLB formation buffer (25 mм Tris‐HCl, 150 mм KCl, 5 mм MgCl_2_, pH 7.5) to 4 µg µL^−1^, and vortex until the lipid films are completely resuspended, forming a cloudy solution containing multilamellar vesicles (MLVs) of various sizes. The obtained large multilamellar vesicle suspensions were then sonicated until solutions were clear. Aliquots were stored at − 20 °C and re‐sonicated before use. SLB formation buffer was used to dilute sonicated small unilamellar vesicle (SUV) aliquots to 0.5 µg µL^−1^ and solutions added into liquid chambers pre‐warmed to 37 °C. Chambers were incubated for 5 min at 37 °C and SLBs were washed ten times with 50 µL SLB washing buffer (25 mм Tris pH 7.5, 150 mм KCl) while cooling down to room temperature.

For experiments with Peltier stage and DNA‐lipid linker chambers were built with a spacer in between two coverslips to form a flat chamber which was closed after SLB formation and filling with 20 µL DNA sample. For UV‐induced wetting, 0.5 µм DNA‐lipid linker were incubated on SLBs for 20 min. To ensure imaging at thermodynamic equilibrium, SLBs were incubated for 16 h at 37 °C.

For experiments involving targeting SUVs to DNA droplets 2 µL of SUV aliquots were used in a final reaction volume wit 5 µм Y‐motifs.

### Inverted Emulsion Transfer

To prepare a lipid‐mineral oil solution 1‐palmitoyl‐2‐oleoyl‐glycero‐3‐phosphocholine (POPC) (Avanti Polar Lipids, Alabaster, AL, USA) and 0.01% of ATTO565 labeled 1,2‐dioleoyl‐sn‐glycero‐3‐phosphoethanolamine (DOPE) (ATTO‐Tech GmbH, Siegen, Germany) were dissolved in chloroform. 50 µL of the 25 g L^−1^ lipid mixture was dried under a N_2_ stream. Lipids were dissolved in 1.5 mL of mineral oil and sonicated for 30 min at elevated temperature (*T* ≥ 40 °C).

GUVs were formed using a 2 mL reaction tube. 250 µl of the lipid‐oil mixture was layered on top of 1 mL outer solution containing condensation buffer and 200 mм glucose. 15 µL inner solution containing condensation buffer, 200 mм sucrose, 10 µм preformed Y‐motifs and 0.5 µm DNA‐lipid linker and 750 µL of the lipid‐oil mixture were mixed in a 1.5 mL tube. The emulsion was added to the multi‐layered solution. Vesicles were obtained by centrifugation for 10 min at room temperature at 6000 × g. After centrifugation, the oil phase was discarded and the vesicle pellet restored in another tube containing fresh buffer. For electrodeformation experiments, glucose and sucrose concentrations were reduced to 100 mм and NaCl concentrations reduced to match an initial osmolarity of 478 mOsm kg^−1^. Vesicle pellets were transferred to a NaCl‐free glucose solution with an osmolarity of 602 mOsm kg^−1^. The osmolarities were measured using an osmometer (Fiske Micro‐Osmometer model120, Fiske Associates, Norwood, MA, USA).

### Electrodeformation

For electrodeformation experiments, sucrose concentration was reduced to 100 mм and NaCl concentration was reduced to 200 mм. Microscope cover slips were passivated with 200 µL of 200 mg mL^−1^ BSA solution for 15 min. Cover slips are washed with 200 µL of ddH2O, then 100 µL of the outer aqueous solution. The electrodeformation chamber consists of a PTFE frame with a central chamber containing two platinum electrodes 1 mm apart connected to copper tape as electrode handles. The chamber is placed on top of the passivated cover slip and filled with 135 µL of outer aqueous solution, followed by pipetting 5 µL of GUVs with a cut pipet tip into the area between the electrodes. The sample is placed on the microscope and connected via the electrode handles to an AC function generator at room temperature. Vesicles larger than 5 µm were deformed by turning on function generator (500 kHz, 10 V). Illumination with 405 nm laser at 100% power for 1 min.

### Microscopy and Activation of DNA‐Lipid Linker

Most images were taken on a Zeiss LSM780 confocal laser scanning microscope using a Zeiss C‐Apochromat × 40/1.20 water‐immersion objective or a Plan‐Apochromat 20x/0.80 air objective (Carl Zeiss). A pinhole size of 2.6–4 Airy units for the channels 512 × 512‐pixel resolution and a pixel dwell time of 1.27 µs was used. For 3D imaging, z‐stacks with 0.2 µm intervals were obtained. For electrodeformation experiments spinning disk confocal imaging was performed using a Nikon/Yokogawa CSU‐W1 spinning disk confocal microscope. A 50 µm pinhole spinning disk was utilized at a rotation speed of 4000 rpm. The samples were illuminated using a Nikon Apo TIRF 60x Oil DIC N2 immersion oil objective. Images were captured with pco.edge sCMOS cameras (pco.edge 4.2 LT USB), set to an exposure time of 100 ms per frame. Z‐stacks were acquired with an optical sectioning set at 0.2 µm intervals.

Both systems were equipped with 405, 561, and 641 nm laser lines to facilitate excitation of different fluorophores. 405 nm laser line was used for photoillumination of DNA‐lipid linker in electrodeformation experiments, 561 nm laser line was used for the membrane dye, and 641 nm laser line was used for the condensates. For all other experiments, DNA‐lipid linker was activated by an illumination system (pE‐4000, CoolLED, UK) after selecting 365 nm at 50% power The equatorial plane of the vesicle and the equatorial plane of the buds were selected manually from the z‐stacks.

### Fluorescence Recovery after Photobleaching (FRAP) Experiment

A defined region of interest (ROI) was selected and bleached using a high‐intensity laser pulse to irreversibly reduce fluorescence in that area. Fluorescence recovery was monitored via time series. The intensity of the bleached region was quantified to generate recovery curves and to extract half‐life.

### Image Processing and Determination of Contact Angles, Heatmaps and Vesicle Surface Area

Microscopy images were processed using NIH Fiji ImageJ. Contrast and brightness adjustments were applied uniformly to the entire image field. The software was also used to quantify surface coverage and contact angles. To quantify the contact angles at the droplet–membrane interface from z‐stacks, two key points at the interface between the droplet, membrane, and surrounding solution were selected at the droplet center. In addition, three more points were manually selected to outline the droplet's perimeter. The contact angles were determined using contact angle plugin for image analysis by applying a spherical fit to the droplet interface.

Concentric circles plugin was used to section GUV images merged over time and in z in five concentric circles in a non‐destructive overlay. Average intensities of condensate channels along the perimeter of each circle are calculated to quantify average condensate positions.

To determine the surface area of GUVs an ellipse was manually fit to the equatorial plane of the vesicle before illumination and the major and minor axes of the ellipse measured. Major/minor axes were used to estimate the surface area of the vesicle before and after illumination. A circle was manually fit to the bud formed after illumination and the diameter measured to estimate the surface area. To estimate the surface area of the vesicle with the bud after illumination, either ½ or ¾ of the surface area of the bud (depending on the degree of protrusion from the vesicle) was added to the vesicle surface area and the surface area of the circle where the bud is joined to the vesicle subtracted (either πr2 in the case of 50% protrusion or 3πr^2/4^ in the case of 75% protrusion).

### Example Calculation for *p*‐Values

To calculate the p‐value, which is defined as the ratio of the penetration depth (d) to the radius (R) of the DNA droplet, the following Equation (3) is used:

(3) $$p=\frac{d_{bud}}{R_{droplet}}$$

where *d_bud_* is the penetration depth, i.e., the distance from the edge of the vesicle to the contact area between the condensate and the membrane and *R_droplet_* is the radius of the DNA droplet. As an example, the penetration depth (d) assumed is 1.5 µm and the radius of the DNA droplet (R) is 1.25 µm.

(4) $$p=\;\;\frac{d_{bud}}{R_{droplet}}=\;\;\frac{1.5\;\mu m}{1.25\;\mu m}=1.2$$

A *p*‐value of 1.2 indicates partial budding of the DNA droplet, as it lies between 0 and 2.

### DNase 1 Treatment and Gel Electrophoresis

For DNA digestion 2.5 mм MgCl_2_ and 0.5 mм CaCl_2_ were added to buffes. Samples were incubated with 1 µL DNase I (Biolabs) per 50 µL reaction volume at 37 °C for 60 min. To examine digenstion a 0.8% agarose gel was prepared by dissolving agarose in 1x TAE buffer and heating until fully dissolved. The solution was poured into a gel casting tray with a comb to create wells and allowed to solidify at room temperature. Once set, the gel was placed in an electrophoresis chamber filled with 1x TAE buffer. DNA samples were mixed with loading dye and loaded into the wells alongside a DNA ladder for size reference. Electrophoresis was performed at 80 V for ≈45 min. The gel was then stained with SYBR Safe and visualized under UV light.

### Statistics

Statistical significance for changes in contact angles of free‐standing condensates on membranes was calculated by one‐way analysis of variance (ANOVA) with Tukey's multiple comparisons. Statistical analysis was performed with GraphPad Prism version 8. 4. and statistical significance is indicated by: ****P ≤ 0.0001; ***P ≤ 0.001; **P ≤ 0.01; *P ≤ 0.05; NS (not significant) *P* > 0.05.

### ChatGPT for Grammar Verification and Translation

The authors used AI‐based language model ChatGPT developed by OpenAI for language optimization and assistance with phrasing in the preparation of this manuscript.

## Conflict of Interest

The authors declare no conflict of interest.

## Author Contributions

N.K. and P.S. conceptualized the work. N.K performed and analyzed most of the experiments, with S.B. and Y.Q. performing and analyzing the electrodeformation experiment. P.S. supervised the research. N.K. wrote the manuscript with contributions from all authors.

## Supporting information



Supporting Information

Supplemental Movie 1

Supplemental Movie 2

Supplemental Movie 3

Supplemental Movie 4

Supplemental Movie 5

Supplemental Movie 6

## Data Availability

The data that support the findings of this study are available from the corresponding author upon reasonable request.
