# Supplementary material for: Designing Tunable DNA Condensates to Control Membrane Budding Transformation in Synthetic Cells
Source: Adv Sci (Weinh). 2025 Jun 27;12(31):e15510. doi: 10.1002/advs.202415510 (PMC12376574; doi:10.1002/advs.202415510)
Supplement: Supplementary file 1 — Supporting Information [file ADVS-12-e15510-s005.docx]

Supporting Information

Designing Tunable DNA Condensates to Control Membrane Transformation

in Synthetic Cells

Nastasja Kaletta, Sophia Burick, Yusuf Qudbuddin, Petra Schwille*

Table S1: Oligonucleotide sequences of Y-motifs. Strand can be modified by cholesterol (/chol), fluorophores ([ATTOX]) or photocleavable group (/iSpPC/). PC_Rev_compl is reverse complement to PC_chol to form a double-stranded DNA-lipid linker. Sticky overhang in bold. The nomenclature orth Y-x_x refers to DNA strands with orthogonal overhangs.

| Name | Sequence (5‘ - 3‘) |
| --- | --- |
| Y-1_6  Y-2_6  Y-3_6  Y-1_6_chol  Y-2_6_ATTO488  Y-2_6_ATTO647  PC_chol  PC_Rev_compl | **CTCGAG**AAAGGAACTCTCCGCGTTGACAAAGCCGACACGT  **CTCGAG**GCCTCTGTGTCGCATCTTCGCGGAGAGTTCCTTT  **CTCGAG**ACGTGTCGGCTTTGTCTTGATGCGACACAGAGGC  **CTCGAG**AAAGGAACTCTCCGCGTTGACAAAGCCGACACGT  [ATTO488]-GCCTCTGTGTCGCATCTTCGCGGAGAGTTCCTTT  [ATTO647]-GCCTCTGTGTCGCATCTTCGCGGAGAGTTCCTTT  CTGCATCCTA/iSpPC/**CTCGAG** AAAGGAACTCTCCGCGTTGACAAAGCCGACACGT/chol  ACGTGTCGGCTTTGTCAACGCGGAGAGTTCCTT/iSpPC/TCTCGAGTAGGATGCAG |
| orth Y-1_6 | **GCTAGC**CAGTGAGGACGGAAGTTTGTCGTAGCATCGCACC |
| orth Y-2_6 | **GCTAGC**CAACCACGCCTGTCCATTACTTCCGTCCTCACTG |
| orth Y-3_6 | **GCTAGC**GGTGCGATGCTACGACTTTGGACAGGCGTGGTTG |
| orth Y-2_6_ATTO488 | [ATTO565]- CAACCACGCCTGTCCATTACTTCCGTCCTCACTG |
| Y-1_4 | **GCGC**AAAGGAACTCTCCGCGTTGACAAAGCCGACACGT |
| Y-2_4 | **GCGC**GCCTCTGTGTCGCATCTTCGCGGAGAGTTCCTTT |
| Y-3_4 | **GCGC**ACGTGTCGGCTTTGTCTTGATGCGACACAGAGGC |


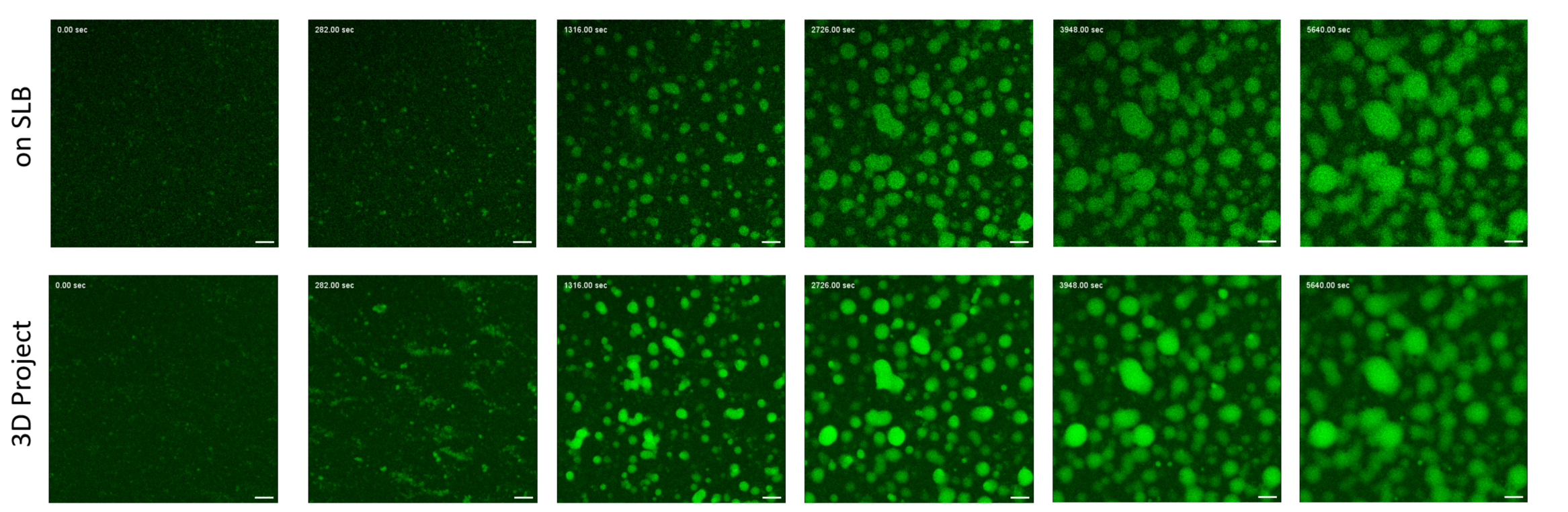


Figure S1: Serial confocal images of droplet-formation on supported lipid bilayers (SLBs) directly on the membrane (a) and as 3D project considering events in z. Scale bar 10 µm.


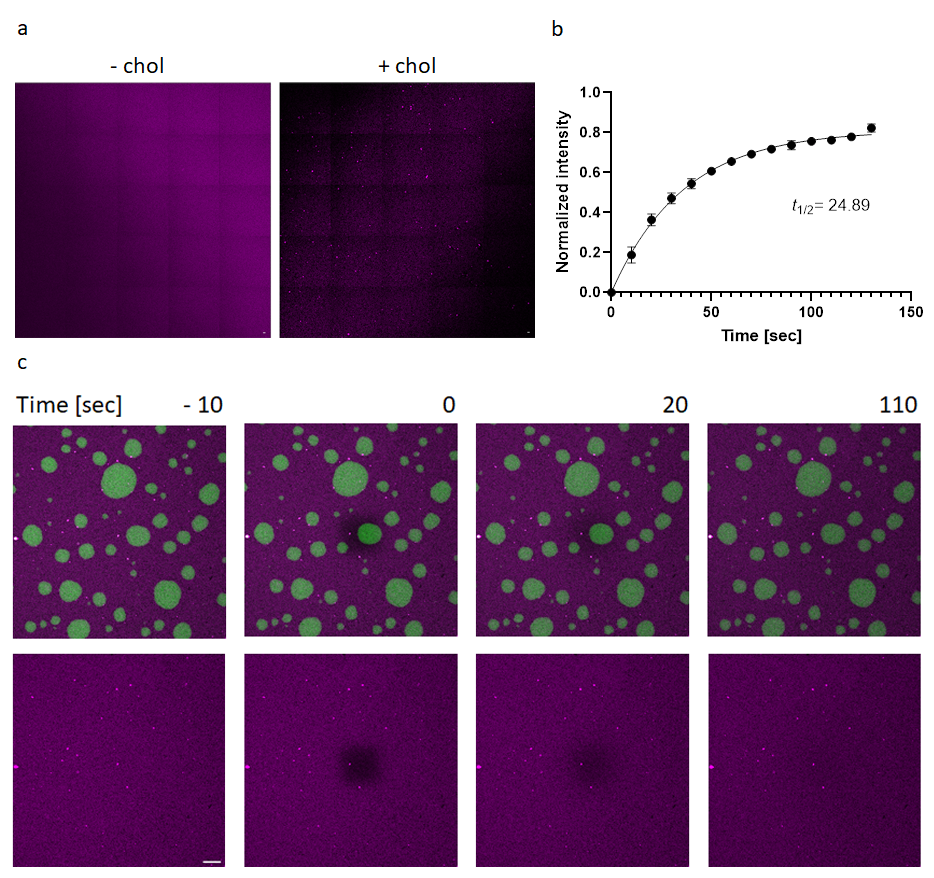


Figure S2: FRAP experiment performed with SLBs wetted by cholesterol-functionalized DNA droplets to characterize the fluidity of the SLBs. a) 10x10 tile scans of SLBs treated as during droplet-formation procedure without (- chol) and with (+ chol) cholesterol-modified DNA strand. b) Quantification of FRAP experiment performed with SLBs wetted by cholesterol-functionalized DNA droplets to characterize the fluidity of the SLBs. Bars show the mean ± SD of measurements n = 3. Half-life indicated in plot. c) Serial confocal images of FRAP experiments shown as merge with DNA channel (ATTO488, top) and membrane channel (DOPE-ATTO 655, bottom) only. Scale bars 10 µm.


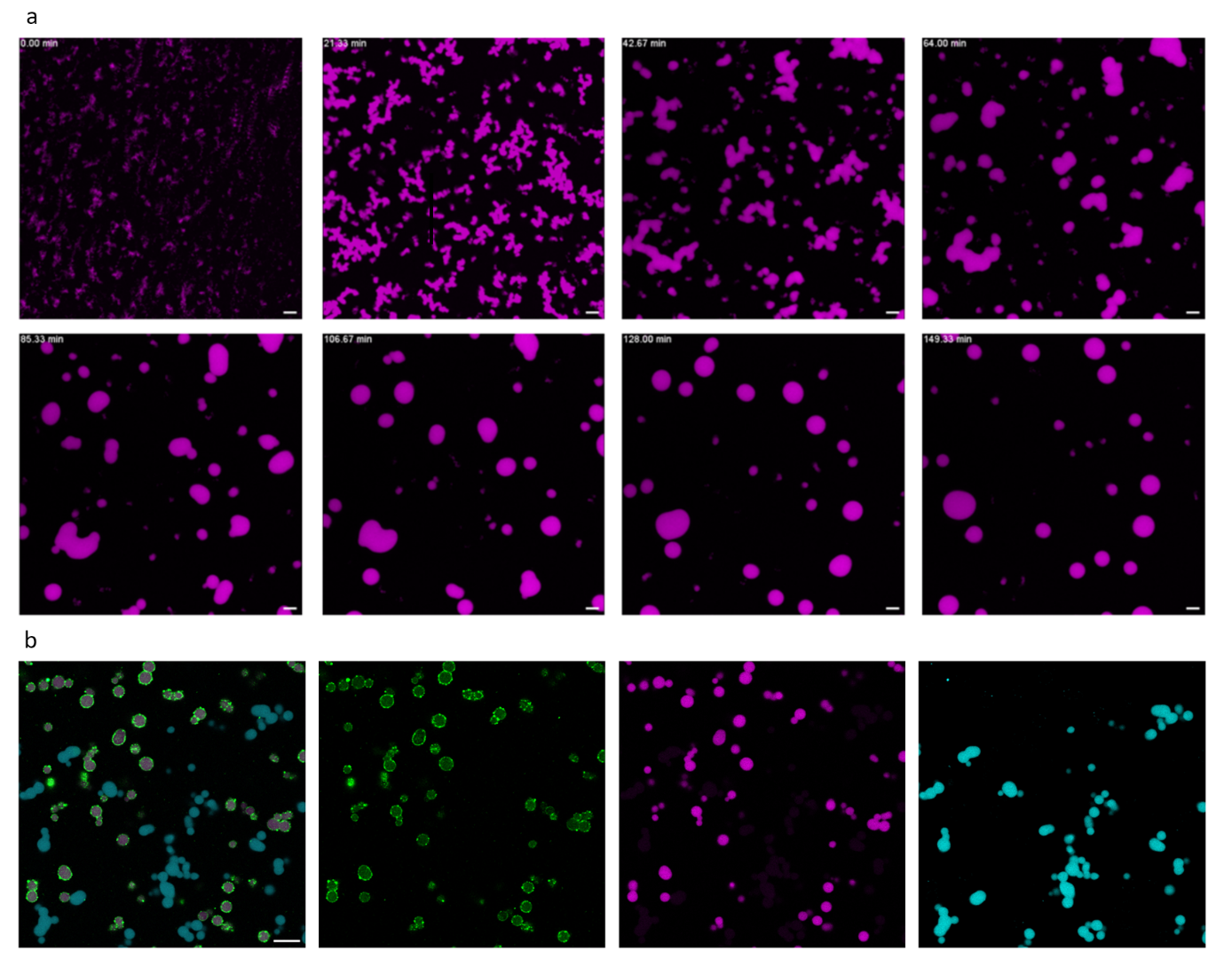


Figure S3: a) Serial confocal images of droplet-formation on DNA-lipid linker functionalized supported lipid bilayers (SLBs) in the inactivated state. b) Confocal images of the merge, SUVs, complementary DNA droplets and, orthogonal droplets were labeled with ATTO 488, ATTO 647, and ATTO 565, respectively. Scale bar 10 µm.


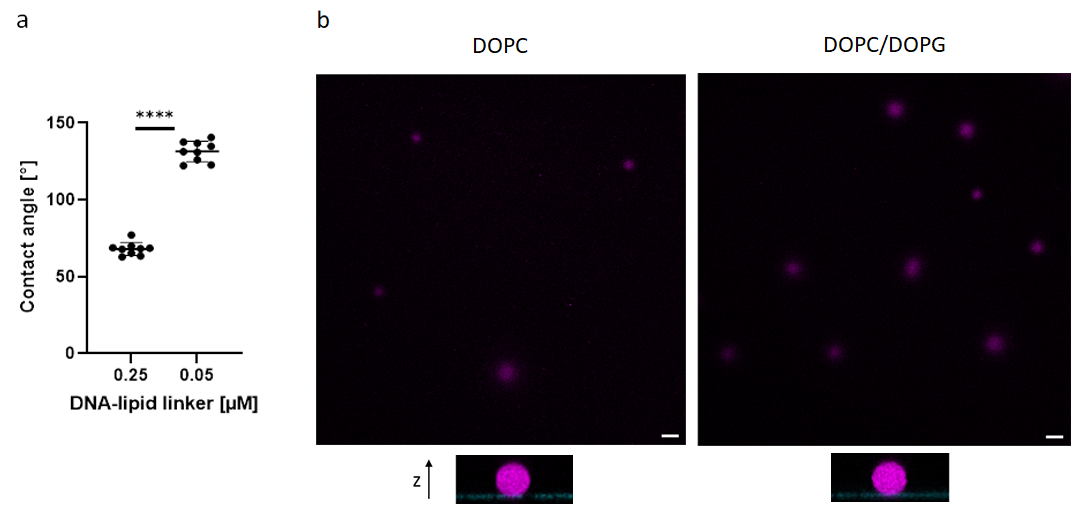


Figure S4: a) Statistical analysis of contact angles for three replicates per different DNA-lipid linker concentrations. Data are represented as mean ± s.d. One-way ANOVA F (3, 32) = 491.7; P < 0.0001; Tukey’s multiple comparisons: *P ≤ 0.0332, **P ≤ 0.0021, ***P ≤ 0.0002, ****P ≤ 0.0001. 5 vs 15 P = < 0.0001; 15 vs 90 P = < 0.0001; 90 vs 300 P = < 0.0001. b) Representative confocal images showing contact areas between DNA droplets and 100 % DOPC membranes (left) and DOPC:DOPG (70:30) membranes with DNA channel (ATTO655) and membrane channel (DOPE-ATTO 565). Scale bars 10 µm.


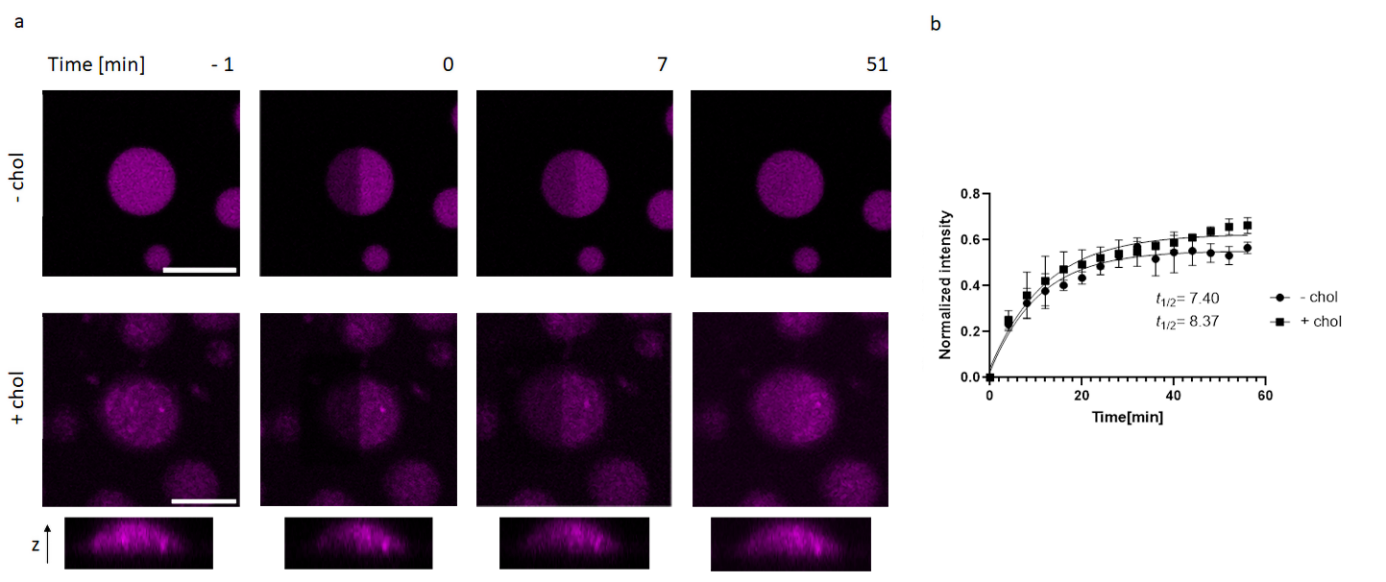


Figure S5: FRAP experiment performed with DNA droplets on SLBs without (- chol) and with (+ chol) DNA-lipid linker. b) Quantification of FRAP experiment performed. Half-life indicated in plot. Bars show the mean ± SD of measurements n = 3. Scale bars 10 µm.


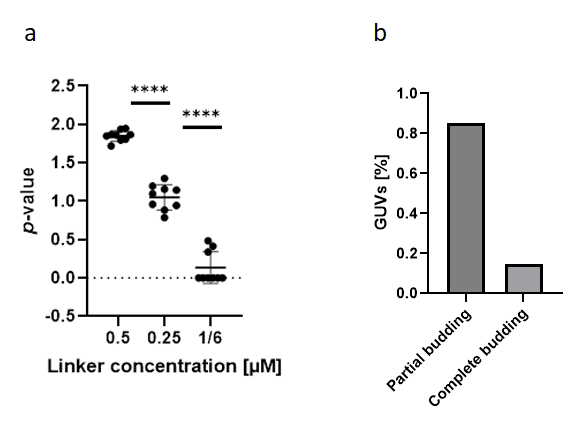


Figure S6: a) Statistical analysis of p-values per different DNA-lipid linker concentrations in GUVs. Data are represented as mean ± s.d. One-way ANOVA F (3, 32) = 491.7; P < 0.0001; Tukey’s multiple comparisons: *P ≤ 0.0332, **P ≤ 0.0021, ***P ≤ 0.0002, ****P ≤ 0.0001. 5 vs 15 P = < 0.0001; 15 vs 90 P = < 0.0001; 90 vs 300 P = < 0.0001. b) Quantification of condensate-induced membrane transformations observed in free-standing GUVs (n = 50).


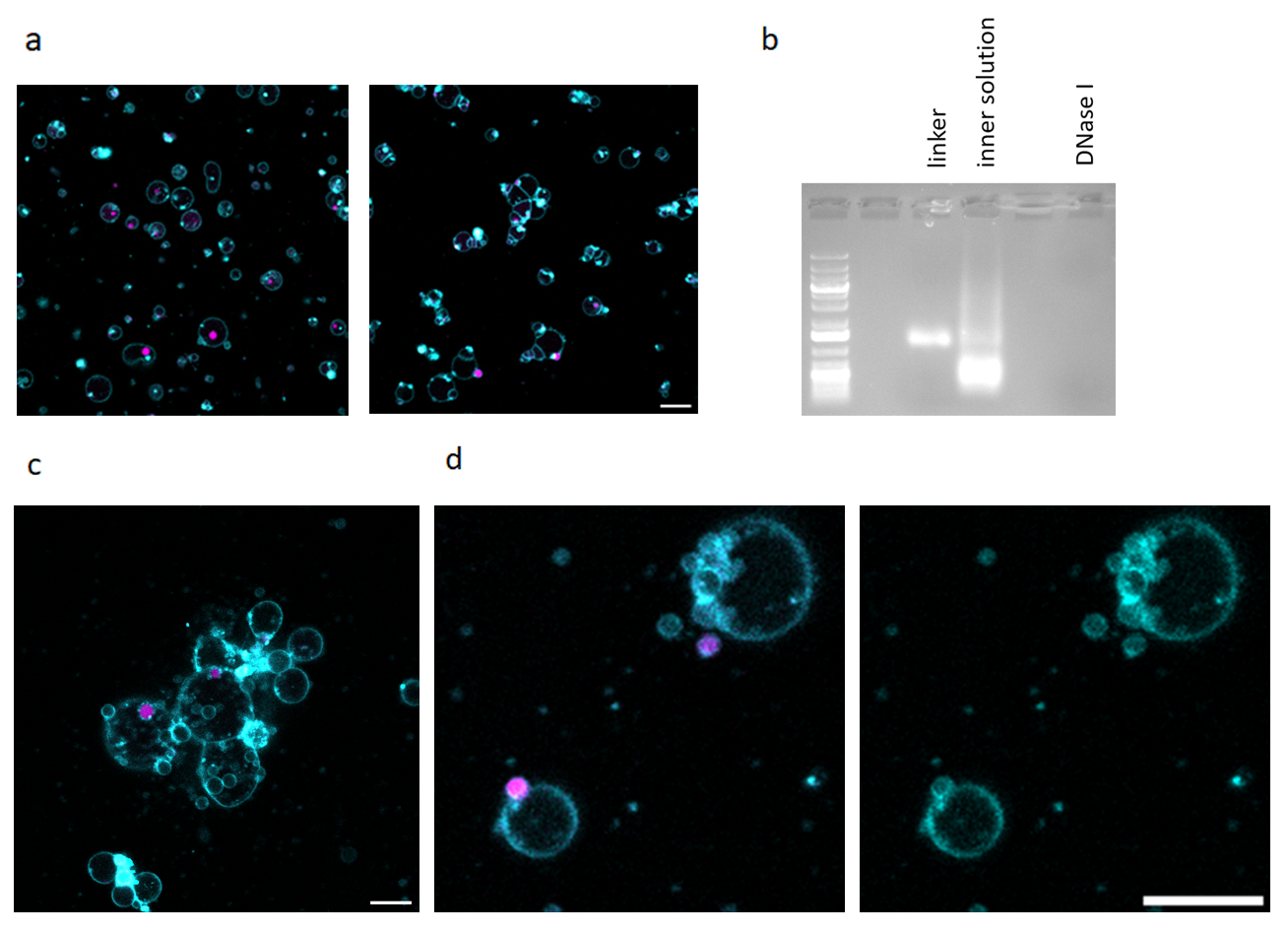


Figure S7: a) Confocal images of GUVs containing DNA-lipid linker and condensates before (left) and after (right, 71 min) UV exposure with DNA channel (ATTO655) and membrane channel (DOPE-ATTO 565). Increased stickiness of GUVs was observed after linker activation. Scale bar 10 µm. b) 0.8 % agarose gel confirming digestion of DNA by DNase I. Staining with SYBR Safe. c+d) Representative confocal images of GUVs after DNase I treatment.
