# Supplementary figures and images for: Designing Tunable DNA Condensates to Control Membrane Budding Transformation in Synthetic Cells
Source: Adv Sci (Weinh). 2025 Jun 27;12(31):e15510. doi: 10.1002/advs.202415510 (PMC12376574; doi:10.1002/advs.202415510)

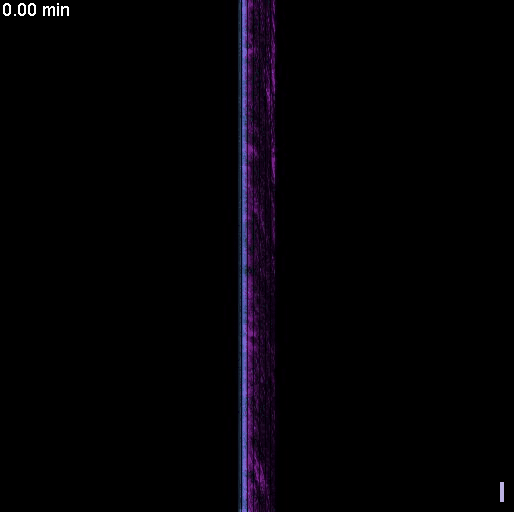

Supplement: Supplementary file 3 — Supplemental Movie 2 [file ADVS-12-e15510-s002.gif]

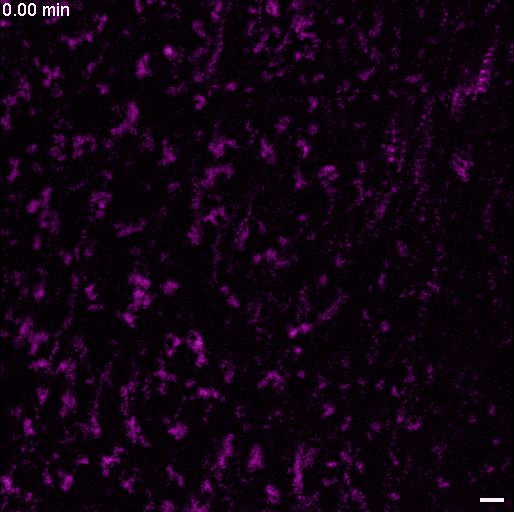

Supplement: Supplementary file 4 — Supplemental Movie 3 [file ADVS-12-e15510-s001.gif]

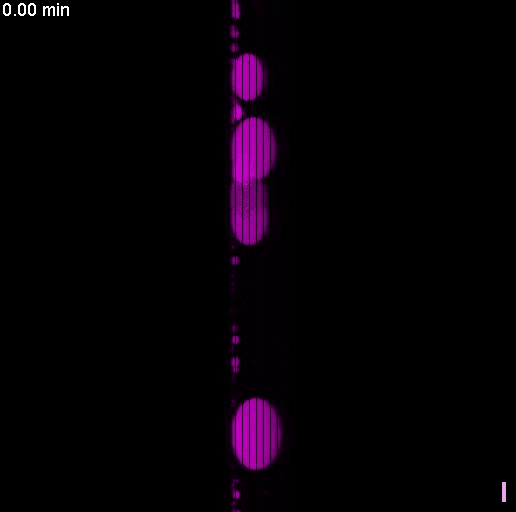

Supplement: Supplementary file 5 — Supplemental Movie 4 [file ADVS-12-e15510-s003.gif]

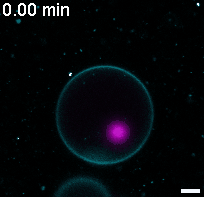

Supplement: Supplementary file 6 — Supplemental Movie 5 [file ADVS-12-e15510-s004.gif]

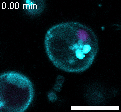

Supplement: Supplementary file 7 — Supplemental Movie 6 [file ADVS-12-e15510-s006.gif]
